# Signaling pathways, microenvironment, and targeted treatments in Langerhans cell histiocytosis

**DOI:** 10.1186/s12964-022-00917-0

**Published:** 2022-12-19

**Authors:** Xue-min Gao, Jian Li, Xin-xin Cao

**Affiliations:** 1 Department of Hematology, Peking Union Medical College Hospital, Chinese Academy of Medical Sciences and Peking Union Medical College, Beijing, 100730 China; 2 State Key Laboratory of Complex Severe and Rare Diseases, Peking Union Medical College Hospital, Chinese Academy of Medical Sciences and Peking Union Medical College, Beijing, China

**Keywords:** Langerhans cell histiocytosis (LCH), MAPK pathway, Inflammatory myeloid malignancy, Progenitor cells, RAF inhibitors, MEK inhibitors

## Abstract

Langerhans cell histiocytosis (LCH) is an inflammatory myeloid malignancy in the “L-group” histiocytosis. Mitogen-activated protein kinase (MAPK) pathway activating mutations are detectable in nearly all LCH lesions. However, the pathogenic roles of MAPK pathway activation in the development of histiocytosis are still elusive. This review will summarize research concerning the landscape and pathogenic roles of MAPK pathway mutations and related treatment opportunities in Langerhans cell histiocytosis.

**Video abstract**

**Supplementary Information:**

The online version contains supplementary material available at 10.1186/s12964-022-00917-0.

## Background

Langerhans cell histiocytosis (LCH) is a histiocytic disorder characterized by abnormal accumulation and differentiation of cells originating from the mononuclear phagocyte system [1]. “Histiocyte”, meaning “tissue cell”, describes cells with mononuclear cell morphologic and immunophenotypic features [2]. LCH is characterized by granulomatous lesions that comprise CD1a^+^ and langerin^+^ (CD207^+^) histiocytes and abundant inflammatory background cells [3]. Those granulomatous lesions could form practically in all organs and tissues, causing copious clinical manifestations, such as osteolytic bone lesions, skin rashes, diabetes insipidus, pulmonary cysts and nodules, and central nervous system involvement [3]. LCH is more common in children, but could also affect adults [3]. In adults, LCH is clinically categorized into the following 4 subtypes: single-system unifocal disease (SS-s), single-system multifocal disease (SS-m), multisystem disease (MS), and single-system pulmonary disease (PLCH) [1]. In children, the involvement of the liver, spleen, and bone marrow are regarded as risk organs (RO) that sub-categorize MS disease into [4] MS-RO+ and MS-RO-. Nearly all LCH lesions carry recurrent mutations in the mitogen-activated protein kinase (MAPK) pathway, with 50% of patients carrying *BRAF* mutations and 25% bearing *MAP2K1* mutations [4]. The phosphorylated extracellular-signal-regulated kinase (ERK) (the downstream of the activated MAPK pathway) was detected in nearly all LCH lesions [5–7], which evinced the general activation of the MAPK pathway in LCH. Thus, LCH is regarded as an inflammatory myeloid malignancy and is grouped as “L-group” histiocytosis together with Erdheim-Chester disease (ECD), highlighting both the clonal and inflammatory properties of LCH [4]. Current front-line therapy for systemic LCH were chemotherapies such as cytarabine and methotrexate [3, 8]. With the evolving understanding of the central role of the MAPK pathway in LCH, effects of MAPK pathway inhibitors are under investigation and have shown promising results. Other treatments, including immunomodulatory drugs [9] and drugs targeting other kinase pathways [10, 11] also provide choices for LCH patients. However, the relationship between the genotype and phenotype of LCH cells, and the function of inflammatory background is still unclear. This review will summarize the understanding of the function of kinase-activating pathways, especially the MAPK pathway, in the pathogenesis of Langerhans cell histiocytosis and related treatment opportunities.

## Recurrent somatic mutations in Langerhans cell histiocytosis

The discovery of nonrandom X-inactivation of CD1a^+^ cells in LCH lesions in 1994 cast the first light on the perpetual malignancy versus inflammation debate on the origin of LCH, suggesting the clonality of LCH cells [12, 13].

With the development of sequencing technologies, Rollins et al were the first to report recurrent *BRAF*^V600E^ mutation in LCH lesions, which was detected in 57% of LCH individuals with OncoMAP and pyrosequencing methods [5], and this finding was later validated in other studies [14–17].

**Fig. 1:**
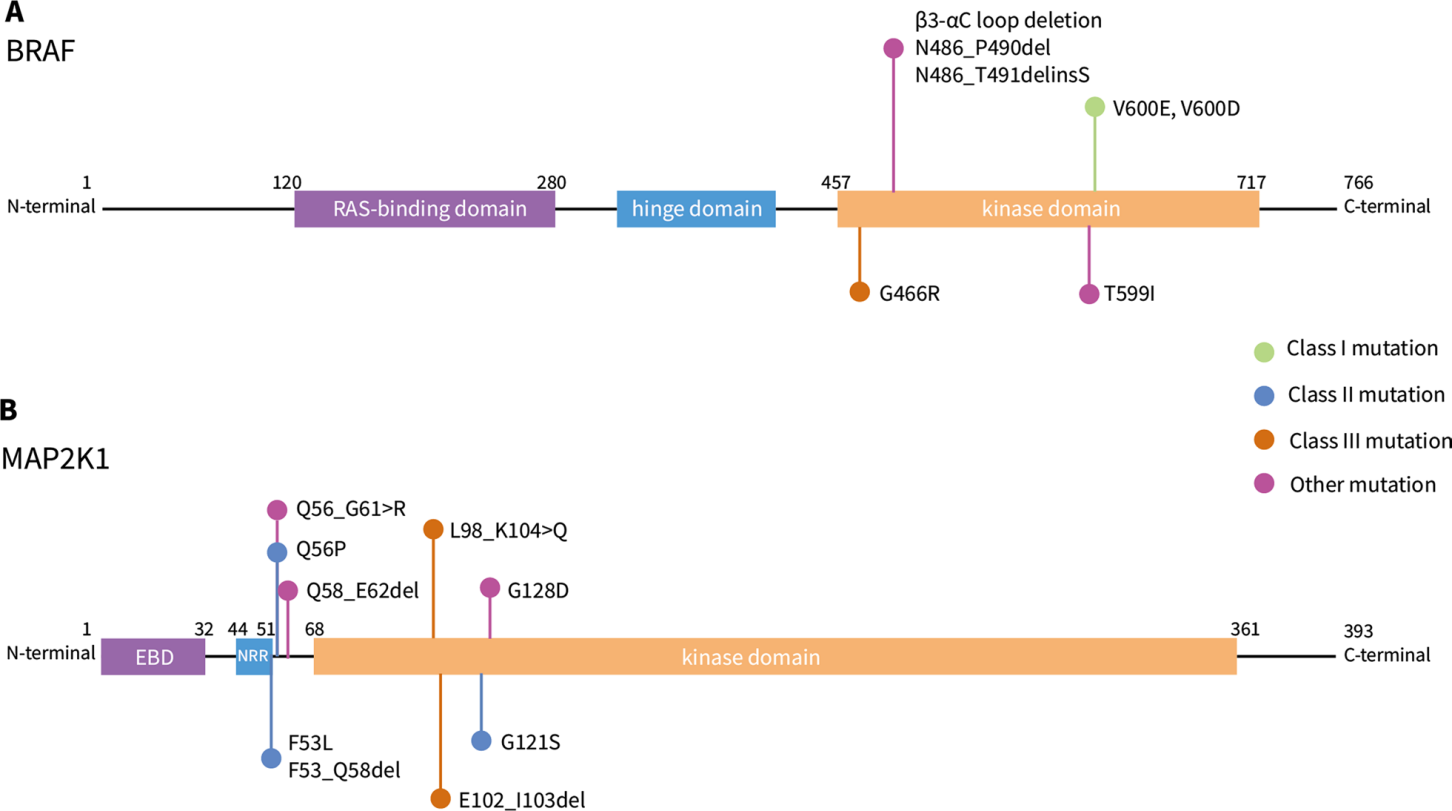
Schematic depicting the domain of *BRAF* and *MAP2K1*, and major mutations identified in LCH. **A** BRAF comprises three main domains, the RAS-binding domain (120–280 amino acids), hinge domain, and kinase domain (457–717 amino acids). V600 mutation belongs to class I *BRAF* mutation, resulting in an activated monomer of the Braf molecule. The N486_P490del mutation results in a shortened β3-αC3 loop of the Braf molecule. **B** MAP2K1 comprises three main domains, the ERK-binding domain (EBD), negative-regulatory domain (NRR), and kinase domain (68–361 amino acids). *MAP2K1* mutations found in LCH were mainly class II (RAF-regulated) and class III (RAF-independent) mutations

**Fig. 2:**
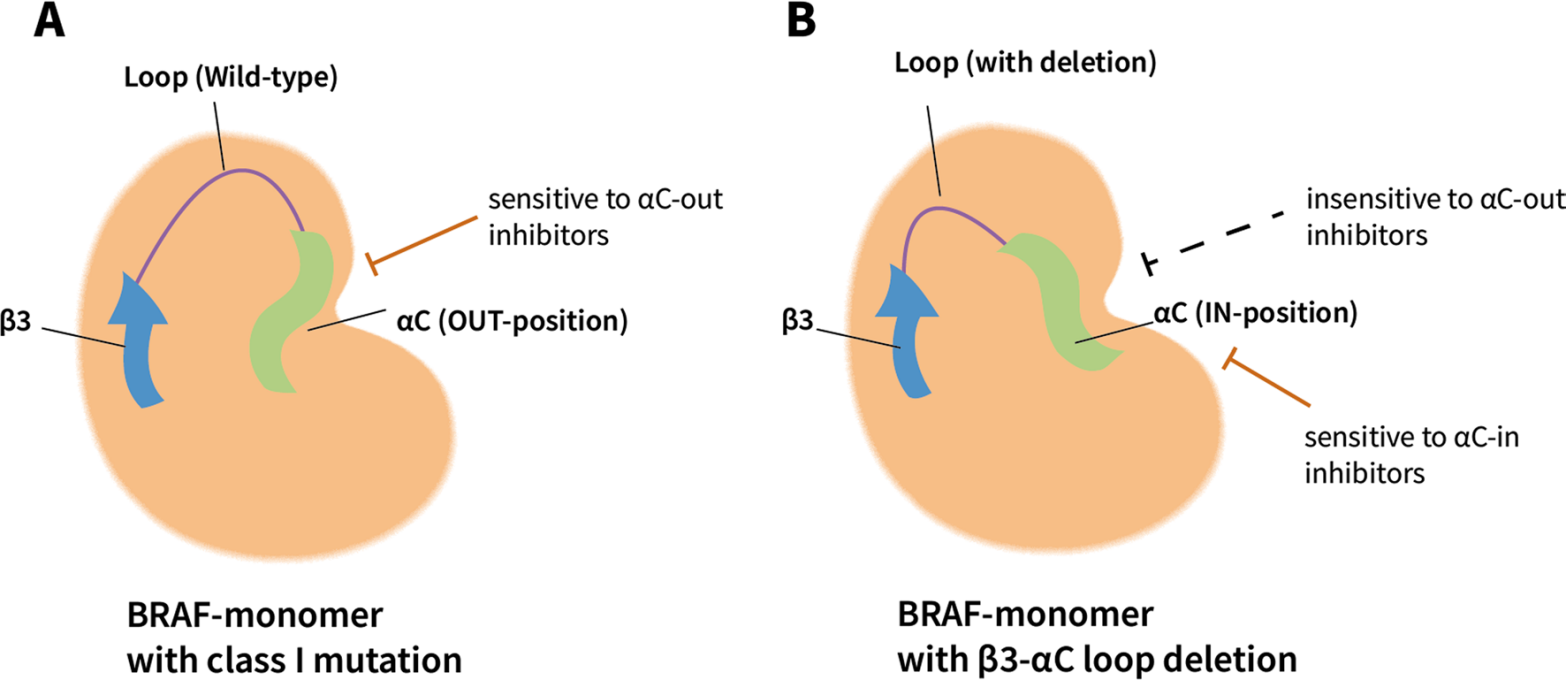
Schematic depicting of BRAF-monomer with different mutation and their sensitivity to BRAF inhibitors. **A** Class I mutations result in an active form BRAF monomer, the αC3 is in the OUT position, making it sensitive to αC-out BRAF inhibitors. **B** The β3-αC3 loop deletion shortens the loop, forced the αC3 in the IN positive, and makes it insensitive to αC-out BRAF inhibitors, but sensitive to αC-in BRAF inhibitors

Mammalian cells include three rapidly accelerated fibrosarcoma (RAF) genes, that is *ARAF*, *BRAF*, and *CRAF*. They are in the MAPK signaling cascade, in which RAF, MEK and ERK1/2 phosphorylate one another sequentially. BRAF is the major RAF kinase that is involved in neoplasms [18]. RAF family protein contains three conserved regions (CR), CR1 includes a RAS GTP-binding self-regulatory domain (RBD), CR2 is a hinge region, and CR3 is the catalytic serine/threonine-protein kinase domain [19]. BRAF contains 766 amino acids, CR1 and CR3 are composed of 120-280 and 457-717 amino acids, respectively (Figure 1A). In physiological states, BRAF is activated and phosphorylated by RAS, forming a side-by-side dimer as the active form. Based on the mechanism of kinase activation and sensitivity to inhibitors, the *BRAF* mutations are categorized into three classes. Class I are V600 mutations that are RAS-independent and function as an active monomer [20]. Class II are RAS-independent, and function as an active dimer that is resistant to vemurafenib [20]. Class III are kinase-inactivating mutations, dependent on upstream stimulation by RAS for activation [21]. The majority of *BRAF* mutation in LCH involve the V600 site. *BRAF*^*V600E*^ mutation belongs to class I mutation, it could induce constitutive activation of the RAF and MAPK signaling pathway that is correlated with the regulation of cell growth, differentiation, and survival. Other *BRAF* mutations have been identified, such as *BRAF*^*V600D*^ [22], *BRAF*^*V600insDLAT*^ [14], *BRAF*^*R506_K506insLLR*^ [23]. Typically, *BRAF* in-frame deletions are detected in LCH (covering the N486 to P490, e.g. *BRAF*^*N486_P490del*^) [7, 24–27], which shorten the β3-αC loop inside the kinase domain. This shorten lock the helix αC in the IN position, prevent it from the inactive OUT position, and result in an activated BRAF monomer, perform a similar function as *BRAF*^*V600E*^ in persistently stimulating downstream pathways [28, 29] (Figure 2). Notably, unlike in children with LCH that has low *BRAF*^N486_P490del^ mutation rate, this mutation occurs in 28% of adult LCH patients, making it the second most common MAPK pathway mutation [25, 26].

In addition, several studies have identified a higher proportion of ERK activation than *BRAF* mutation [5, 6, 30–32], prompting the exploration for additional activating mutations in the MAPK pathway. Further studies revealed *MAP2K1* mutations in 27.5–33% of *BRAF*^WT^ LCH patients [6, 24, 32–34]. *MAP2K1* encodes mitogen-activated protein kinase kinase 1 (MEK1), which is the downstream of the RAF family in the MAPK pathway. MEK1 compose of an ERK binding domain, an inhibitory segment, and a protein kinase domain (68–361 amino acids) that contains a β3-αC loop area like in all protein kinases [35] (Figure 1B). The protein kinase domain also contains a proline-rich area between 270 and 307 residues that is important for RAF binding [35]. In LCH, mutations in *MAP2K1* frequently occur near the β3-αC loop area, which is a negative regulator of MEK [6], resulting in the activation of MEK [36] and downstream ERK [6, 24, 37]. Rosen et al. revealed that mutations in *MAP2K1* could be classified into three classes based on the dependence of mutated MEK1/2’s phosphorylation on RAS/RAF [38], that is, RAF-dependent (class I), RAF-regulated (class II), and RAF-independent mutations (class III). RAF-dependent *MAP2K1* mutations have little or no ability to activate downstream ERK on their own and often coexist with other activating mutations in RAS or RAF. In contrast, RAF-independent mutations are often indels occurring inside the β3-αC3 loop area, and this type of mutation usually causes hyperactivation, activating downstream ERK on its own, and is unresponsive to current MEK inhibitors. Finally, regarding RAF-regulated *MAP2K1* mutations, mutations in different sites of MAP2K1 have diverse abilities to activate downstream ERK on their own, which is inversely associated with the dependency on RAS- or RAF-activating mutations [38]. *MAP2K1* mutations in LCH were reported to occur in a mutually exclusive manner with *BRAF* mutations [6]. However, class II and III *MAP2K1* mutation were detected in LCH lesions. There were two Rosai-Dorfman disease (RDD) patients carry *BRAF* and *MAP2K1* mutation simultaneously [25]. Whether *BRAF* and *MAP2K1* mutations occur in a mutually exclusive manner in LCH needs further validation.

In a much smaller proportion of patients, other mutations that activate the MAPK pathway were identified. Mutations in another *RAF* family gene, *ARAF*, were also discovered in purified LCH cells [6, 27, 31, 39], but their function requires further confirmation. Mutations in *MAP3K1*, which encodes ERK, were also detected in LCH lesions, including *MAP3K1*^E1286V^ and two truncation mutations (*MAP3K1*^T779fs^ and *MAP3K1*^T1481fs^) [37]. The *MAP3K1*^E1286V^ is likely a germline polymorphism that is fully functional, but the *MAP3K1*^T1481fs^ truncation mutation seems to cause loss-of-function of *MAP3K1*, and the patients carry *MAP3K1*^T779fs^ also carried *BRAF*^V600E^ mutation [37]. Other mutations in MAPK pathway that concurrent with *BRAF*^V600E^ in histiocytosis were also reported, one LCH patients carries *BRAF*^V600E^, *MAPK11*^D277Y^, and *MAP3K9*^R303L^, another ECD patients carries *BRAF*^V600E^ with *MAPK9*^D395Y^ and *MAP3K19*^R132M^, respectively [39]. Mutations in *NRAS* and *KRAS*, which encode RAS family proteins that are upstream of BRAF, were detected in LCH [24, 26, 32]. The mutation of *ERBB3* were also detected in LCH [6]. *ERBB3* encodes a membrane-bound receptor tyrosine-protein kinase ErbB-3, which would activate MAPK and phosphatidylinostitol-3-kinase/protein kinase B (PI3K/Akt) pathway after ligand binding induced receptor dimerization [40]. Another receptor that is upstream of MAPK and PI3K/Akt pathway is colony stimulating factor 1 receptor (CSF1R). *CSF1R* mutation was detected in 2 of 78 LCH patients, 1 of 5 mixed LCH/ECD patients [25] and in other histiocytosis patients [10, 25, 39]. *CSF1R* encodes macrophage colony-stimulating factor (M-CSF) receptor, which is normally expressed on myeloid progenitors, monocytes, macrophages, dermal Langerhans cells, and immature dendritic cells [41, 42]. The activation of CSF1R by its ligand would stimulate the proliferation and differentiation of monocytes via activating MAPK pathway or PI3K/Akt pathway [41]. The above mentioned 2 LCH patients, 1 patient carries *CSF1R*^G936S^ and *BRAF*^N486_P490del^ mutation concurrently, and another patient carries CSF1R^V729G^ and *MAP2K1*^L98_K104delinsQ^ mutation concurrently [25]. Although the function of *CSF1R* mutations in the two LCH patients were not validated, they may activate the MAPK pathway with concurrent *BRAF* or *MAP2K1* mutation in synergy. Bearing two driver mutations in one signaling pathway seems rare in LCH, partly because the target sequence covering the whole signaling pathway is not a routine. Thus, it is recommended in the future for identifying more concurrent driver mutations, which would help reveal the pathogenesis. Fusions involving the *BRAF* and *ALK* genes identified via transcriptome sequencing were also reported in LCH [7, 24, 36, 39].

In addition to mutations affecting the MAPK pathway, mutation of *PIK3CA*, which encodes PI3K in the PI3K-AKT-mTOR (mTOR: mammalian target of rapamycin) pathway, was discovered in 1 (1.2%) of 86 LCH patients [43]. The patient carried the *PI3KCA*^E542K^ mutation, which affects the p110α subunit, preventing the kinase activity of p110α from inhibiting the p85–p110α interaction, leading to constitutive activation of the PI3K-mTOR pathway [43, 44].

Especially, in isolated pulmonary LCH, which is clearly associated with cigarette smoking and can spontaneously regress after smoking cessation, Mourah et al. reported recurrent *BRAF*^*V600E*^ and *NRAS*^*Q61K/R*^ mutations were detected in 50% and (11 of 26) 40% of pulmonary LCH lesions, respectively [32]. Jouenne et al. also reported MAPK pathway mutations detected in (44 of 50) 88% of pulmonary LCH lesions [26]. These results indicate a clonal nature of pulmonary LCH.

**Fig. 3:**
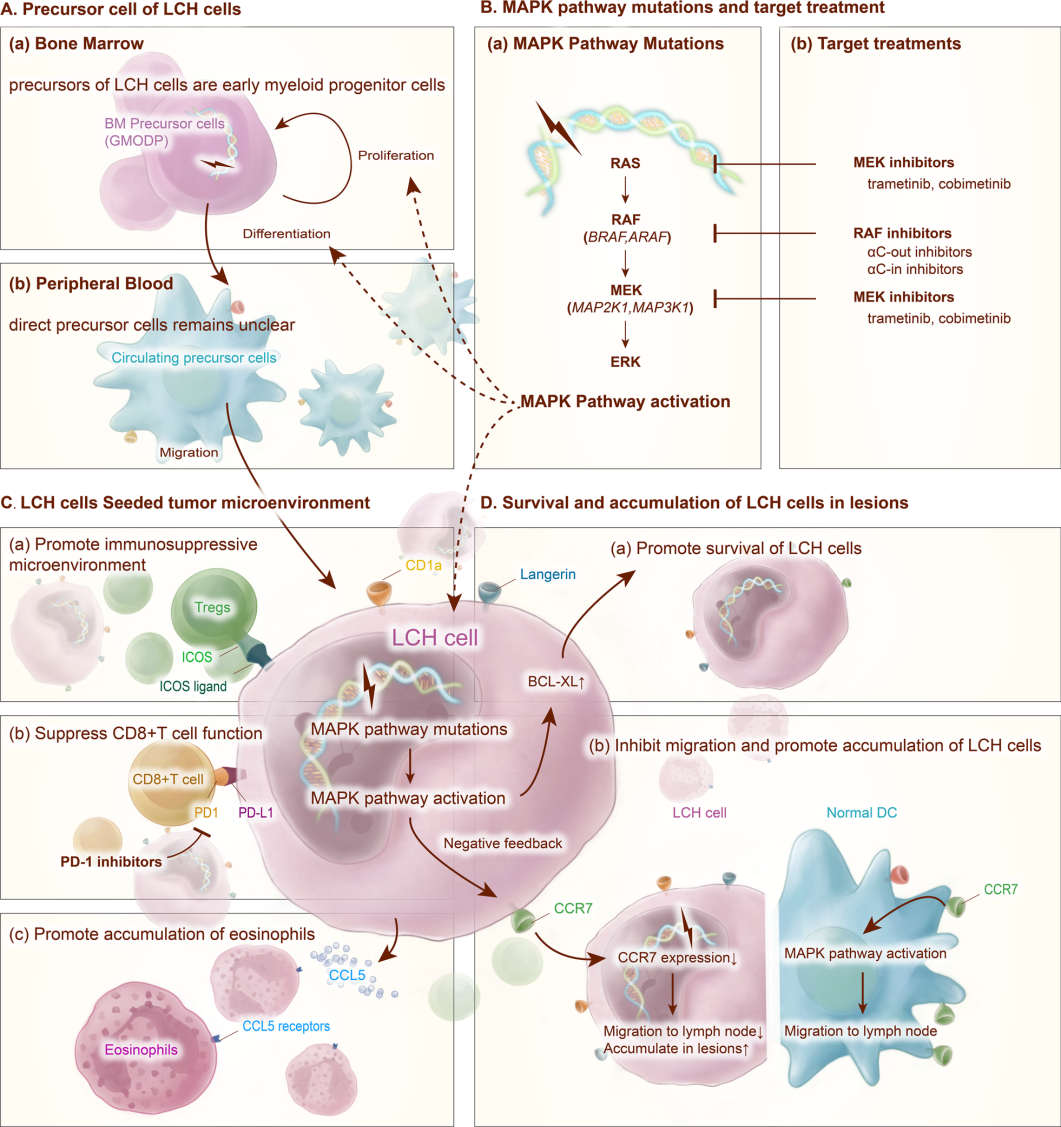
Signaling pathways, microenvironment, and targeted treatments in Langerhans cell histiocytosis. **A** The precursor cells of LCH cells. (a) Early myeloid progenitor cells are the precursor cells of LCH cells, regardless of the risk of disease. MAPK pathway activation promotes the proliferation and differentiation of precursor cells. (b) The direct precursor cells of LCH cells in peripheral blood are still controversial. **B** MAPK pathway mutations and treatments targeting specific mutations. **C** LCH cells seeded the tumor microenvironment. (a) The interaction between LCH cells and Tregs via ICOS-ICOS ligand promotes the accumulation of Tregs in the microenvironment, which promotes an immunosuppressive microenvironment. (b) The interaction between LCH cells and CD8^+^ T cells via PD-1 and PD-L1 suppresses CD8^+^ T cell function and helps LCH cells escape immunosurveillance. PD-1 inhibitors act in synergy with MAPK inhibitors. (c) CCL5 produced by LCH cells promotes the accumulation of eosinophils in lesions via chemotaxis. **D** Survival and accumulation of LCH cells induce the formation of lesions. (a) The activation of the MAPK pathway in LCH cells upregulates BCL-XL expression in LCH cells and promotes the survival of those cells. (b) The activation of the MAPK pathway in LCH cells downregulates CCR7 expression on the cell membrane via negative feedback, inhibits the migration of LCH cells to draining lymph nodes, and promotes LCH cell accumulation in lesions

These results demonstrate a central role of ERK activation in LCH pathogenesis. Mutations in the MAPK pathway, especially mutations in *BRAF* and *MAP2K1*, which account for nearly 80% of mutations in patients, are considered driver mutations in Langerhans cell histiocytosis (Figure 3B(a)).

## Gene expression analysis revealed heterogeneity of LCH cells and potential progenitor cells of pathogenic histiocytes

Driver mutations cause malignancies by affecting transcription and expression processes and utilizing intrinsic signaling pathways in specific host cells, thus revealing that understanding the progenitor cell is vital for understanding the pathogenic role of MAPK pathway activation in LCH. LCH cells share characteristic markers (i.e., CD1a and langerin) with normal epidermal Langerhans cells; thus, malignant transformation of normal epidermal Langerhans cells into LCH cells has been suggested. However, by comparing the expression profile of CD207^+^ cells from LCH lesions with that of control skin CD207^+^ cells, noticeable discrepancies were revealed [45, 46], indicating that LCH cells differ from skin Langerhans cells. They also revealed that expression profile of LCH cells resemble immature conventional dendritic cells (cDC) [45], differ from that of cDC1s and plasmacytoid DCs (pDCs) [46]. Subsequently, by bulk transcriptome analysis, Diamond et al. also demonstrated that the transcriptome of LCH cells resembles those of cDCs (e.g., expression of *IRF7*, *RUNX3*, and *CCR7*) and late-stage myeloid progenitor cells [36]. These results were validated and extended by a landmark single-cell RNA sequencing study by Gao et al., which analyzed CD1a^+^ CD207^+^ cells from LCH lesions, indicating that the gene expression of LCH cells was enriched in neoplastic pathway (e.g., cell cycle, MYC-associated gene, and DNA repair) and inflammation pathway (e.g., inflammation, cellular response to interferon, and antigen processing and presentation) [47]. Furthermore, 14 subsets of LCH cells in LCH lesions were recognized, and a continuous developmental hierarchy of LCH cell *in situ* differentiation was mapped. In the least differentiated cell subsets, gene expression was enriched in DNA replication and cell cycle regulation (including *MKI67*, which encodes Ki-67), indicating the proliferative nature of those cells, those cells could be identified by immunohistochemistry staining of Ki-67 and CD1a [47]. In contrast, in the putative mostly differentiated cell subsets, gene expression was enriched in cytokine signaling and osteolysis, and was similar to that in mature dendritic cells [47]. In addition, the researchers explored the gene accessibility, gene regulators, and gene regulatory networks among the cell subsets, intending to reveal relationships with the MAPK pathway. However, the gene expression profile and regulatory mechanisms were not related to the *BRAF*^*V600E*^ mutation or other MAPK pathway genes [47]. Shi et al also revealed the heterogeneity of LCH cells from LCH patients’ skin by single-cell RNA-seq, the LCH cells with cell proliferation identity (expressing *MKI67* and *CENPF*) were also revealed [48]. They also reported another LCH cell subsets with MPAK pathway gene (*MAPK14*, *MAPK9*, *RRAS*, and *JAK2*) upregulation, with inflammation-related and collagen degradation-related pathway enrichment, and this subset is correlated with cDC2/DC3 (a group of cells including cDC2 and DC3) from LCH patients' peripheral blood [48].

At the protein expression level, immunohistochemical studies also revealed heterogeneity in the LCH cell population. The CD1a^+^ population expresses various intensities of CD207 [47, 49]. In addition, in LCH lesions, multiple myeloid cells are present, including monocytes, macrophages, dendritic cells, and osteoclast-like multinucleated giant cells (MGCs) [50]. The VE1 protein (the *BRAF*^V600E^ gene product) was not only expressed in LCH cells with various expression levels, but also expressed in CD207^−^CD14^+^CD36^+^ monocytes and MGCs, indicating that the *BRAF*^*V600E*^ mutation occurs in a population of myeloid cells with different maturation statuses [15, 51]. Those evidence also demonstrate that LCH cells may develop from immature myeloid cells.

In addition, Hutter et al revealed that Jagged2 (*JAG2*) and *NOTCH1* are highly expressed in LCH cells via transcriptome analysis, and by immunohistochemical study, they proved that the Notch pathway is also activated in LCH cells [46]. JAG2 directly activated the Notch pathway in CD14^+^ monocytes [52], and those cells can also acquire CD1a and langerin expression under stimulation by Jagged2 and Transforming growth factor-beta (TGF-β) [46]. The transcriptional profile of those JAG2-stimulated CD14^+^ monocytes was most similar to LCH cells, compared with CD1a^+^langerin^+^ cells derived from CD14^+^ monocytes (under IL-4, granulocyte macrophage colony-stimulating factor (GM-CSF), and TGF-β stimulation), or derived from CD1c^+^ DCs [52]. The most enriched pathways were associated with inflammation, bone resorption, and granuloma formation [52]. Those cells could also induce proliferation of Tregs and produce a similar cytokine profile [52]. Thus, JAG2 mediated Notch pathway activation in CD14^+^ monocytes contribute to phenotypic and functional resemblances to LCH cells. Thus, Schwentner et al proposed that CD14^+^ monocytes could differentiate toward LCH cells when Notch pathway is activated by JAG2 in certain niches [52].

Another single-cell transcriptomic study focused on the mononuclear phagocytes (MNPs) in peripheral blood from LCH patients, CD14^+^ monocytes, CD16^++^ monocytes, pDCs, cDC1, and cDC2 were identified [48]. All subsets showed significantly higher levels of MAPK-pathway related gene expression than health control, and the signaling activities measured by single-sample gene set enrichment analysis (ssGSEA) were associated with higher cell-free *BRAF*^V600E^ levels [48]. After dabrafenib treatment, inflammation and MAPK signaling pathway genes were downregulated in those peripheral blood mononuclear phagocytic cells [48].

Taken together, these results suggest that LCH cells are likely to arise from bone marrow-derived progenitors.

## Define the precursor of LCH cells and reveal the pathogenic role of MAPK pathway activation

### The origin of CD1a+CD207+ cells in tissues is heterogeneous under an inflammatory state

Epidermal Langerhans cells are members of dendritic cells, together with Kupffer cells and microglia, they were derived from common progenitors from prenatal yolk sack and fetal liver [53]. Dendritic cells in other tissues, however, are derived from circulating dendritic cells that differentiated from bone marrow-derived myeloid progenitor cells [54]. Under normal conditions, circulating dendritic cell progenitors migrate into tissue and become immature dendritic cells. After phagocytizing antigen, they become mature dendritic cells, upregulate maturation markers such as C–C chemokine receptor type 7 (CCR7), CD86, and CD80, migrate into draining lymph nodes [55], and then undergo apoptosis after activating T cells [56]. Under inflammation, circulating monocytes and dendritic cells can be recruited into lesion tissue and differentiate into Langerhans cell-like cells [52, 57–59]. CD1c^+^ dendritic cells can express CD1a and langerin under the stimulation of Thymic stromal lymphopoietin (TSLP) and TGF-β, or GM-CSF and bone morphogenetic protein-7 (BMP7) [57]. CD14^+^ monocytes can express CD1a and langerin under the stimulation of GM-CSF, IL-4, and TGF-β [58]. As mentioned above, CD14^+^ monocytes can also acquire CD1a and langerin expression under stimulation by Jagged2 and TGF-β [46]. CD1a^+^langerin^+^ cells derived from CD14^+^ cells (stimulated by Jagged2 and TGF-β) had a surface marker profile most similar to LCH cells compared with those cells derived from CD1c^+^ DCs [52].

However, the understanding of the control of fate determination in and the differentiation of myeloid cells is continually expanding. Both monocytes and dendritic cells are comprised of heterogeneous subgroups [60] and are derived from hematopoietic stem cells in a not yet well-defined trajectory. In addition, abnormal continuous MAPK pathway activation may induce different differentiation potentials and expression of different surface markers in myeloid cells, which increases the challenges in research.

### Surface markers failed to identify progenitor cells

The most straightforward way to identify a cell is via specific surface markers. However, whether CD1a^+^CD207^+^ cells circulate in peripheral blood or are in the bone marrow is controversial. These cells are detectable with flow cytometry in bone marrow in adults with bone marrow infiltration, whereas they are not detectable in other situations [49, 61]. Given the results from the previously described RNA sequencing study [47], it is suspected that histiocytes originate from precursors that differentiate into Langerhans cell-like cells in situ. However, no phenotypic identifier has been found before their expression of the characteristic surface maker of LCH cells.

### Tracing recurrent mutations for progenitors of LCH cells

Fortunately, recurrent mutations provide us with a molecular tag for tracing the ontogeny of pathogenetic histiocytes. Studies evaluating the mutational state of cells from lesions, peripheral blood, and bone marrow have provided multifaceted evidence and hierarchies for inferring the origin of progenitor cells. In 16 children with LCH with multiple lesions, the *BRAF* genotype was identical in different lesions in the same individual, indicating that LCH histiocytes from different lesions originate from a common progenitor cell [62].

In the peripheral blood of LCH patients, the *BRAF*^*V600E*^ mutation was detectable in CD11c^+^CD1c^+^ dendritic cells, CD14^+^ classic monocytes and CD14^−^CD16^+^ non-classic monocytes almost exclusively in patients with MS disease, correlating with high-risk and active disease, but could not be detected in unfractionated peripheral blood mononuclear cells (PBMCs) from SS-LCH patients [49, 61, 62]. In CD34^+^ cells from bone marrow, *BRAF*^*V600E*^ could also be detected in patients with high-risk or multisystem disease [45, 61]. These evidence led Allen *et al*. to propose a “misguided myeloid differentiation” model in which the differentiation state of the cells acquiring MAPK pathway activation determines the severity of the disease [3]. In this model, MAPK pathway mutations are acquired in bone marrow-resident hematopoietic progenitors in MS-RO+ patients, whereas in low-risk patients, MAPK pathway mutations are acquired in circulating blood cells, and in patients with a single lesion, the mutation occurs in tissue-resident dendritic cells.

However, Collin et al. reported that the *BRAF*^*V600E*^ mutation could also be detected in the peripheral blood of a patient with only one skin lesion, and *BRAF*-mutated cells were detected at a higher level in the peripheral blood of patients with MS-LCH than single system LCH [61]. Furthermore, in a recent study, the *BRAF*^*V600E*^ mutation was detected in dendritic cells, monocytes, B cells, and T cells from peripheral blood of low-risk or single-lesion patients, which supports that an earlier progenitor in the lymphoid-primed multipotent progenitor (LMPP) stage exists for such patients [51]. Combining with the differential hierarchy of LCH cells identified with single-cell RNA sequencing [47], instead of the “misguided myeloid differentiation” model, Borst et al. proposed a “progenitor recruitment and in situ differentiation” model, which supports the idea that in both high- and low-risk LCH patients, LCH cells are derived from oligopotent progenitor cells in the bone marrow that are recruited into inflamed tissues and that the different severities of disease originate from a probability event that correlates with the number of progenitor cells in peripheral blood [51]. This new model can also explain the development of single-lesion disease into multisystem disease in 10% of patients (Figure 3A(a)).

Xenograft experiments were also performed. Durham *et al* extracted CD34^+^ cells from 3 ECD/LCH mixed disease patients and 5 ECD patients, then injected them into irradiated NSGS (nonobese diabetic severe combined immunodeficient gamma with both IL2Rgamma knockout and transgenic expression of human IL-3, stem cell factor and granulocyte-macrophage colony-stimulating factor) mice [63]. Four mice receiving cells from ECD patients had human CD45^+^ cells detected, among which only 1 mouse had ECD phenotype, and no cells from the mixed disease patients were successfully engrafted [63]. Later, Lee et al. transplanted whole bone marrow from infants with *BRAF*^V600E^ mutant LCH and secondary hemophagocytosis into immunodeficient mice, those mice exhibit disease phenotype like human disease [64]. When NSG (nonobese diabetic severe combined immunodeficient gamma) mice were reconstituted with human CD34^+^ cells carrying *BRAF*^V600E^, LCH-like phenotypes developed [65]. Taken together, those evidence demonstrate that LCH cells originate from bone marrow early progenitor cells.

### The direct progenitor cells of LCH cells in peripheral blood remains undetermined

Although the most upstream progenitors of LCH cells are now widely accepted as bone marrow progenitor cells, the exact progenitor cell that carries the mutation in the circulating blood that directly migrates to lesions and then undergoes irreversible differentiation into an LCH cell remains to be determined. (Figure 3A(b)).

### MAPK pathway activation plays different roles in early hematopoietic progenitors and more differentiated histiocytes

The effect of MAPK pathway activation on cell differentiation or fate decisions is still elusive. In early progenitor cells, MAPK pathway activation seems to limit the cell-renew and expansion ability of CD34^+^ hematopoietic cells. *BRAF*^V600E^+ CD34^+^ cells showed a senescence transcriptome (*CDKN2A*, *CDKN2C*, *CDKN2D*, *CD9*, *MDM2*, *MMP*), and phenotype including senescence-associated β-galactosidase (SAβGal) and senescence-associated secretory phenotype (SASP) that could lead to the production of inflammatory cytokines such as IL-1, IL-6, and matrix metalloproteinases (MMPs) [65]. The senescence state could also prolong the survival of those cells via the expression of anti-apoptotic proteins B-cell lymphoma-extra large (BCL-XL) and B-cell lymphoma 2 (BCL-2) [65]. Simultaneously, *BRAF*^V600E^ expression on CD34^+^ hematopoietic cells promote their differentiation towards MNPs and promote granulocyte-macrophage progenitor cell (GMP) proliferation. *BRAF*^V600E^ induced a senescence transcriptome and phenotype in CD34^+^ hematopoietic cells, limiting their self-renew and proliferation ability, but are preferentially differentiated into GMPs, leading to the expansion of GMPs and their offspring [65]. The differentiation skewing toward GMP of *BRAF*^V600E^ CD34^+^ cells was driven by cell-intrinsic mechanisms, the expression profile of those cells was enriched in cDCs and macrophage commitment (*BATF3*, *IRF4*, *CSF1R*, *CLEC10A*), and the granulopoiesis genes were reduced (*ELANE*, *MPO*, *PRTN3*, *CSF3R*) [65]. The differentiation skewing is also influenced by the secreted molecules by themselves, supernatant from *BRAF*^V600E^+ CD34^+^ cells’ culture promotes the BRAF^WT^ CD34^+^ cells to differentiate toward MNPs [65]. Indeed, the frequency of granulocyte/macrophage/osteoclast/dendrite cell progenitor ((G)MODP) cells was significantly higher in LCH patients (regardless of risk status) than in age-matched healthy controls, and this increased frequency correlated with higher disease activity [51]. In cell culture, (G)MODP cells from LCH patients produce more offspring under LC differentiation conditions (TGF-β and TNF-α) than those from healthy controls, suggesting that the *BRAF*^*V600E*^ mutation promotes the proliferation of (G)MODP offspring cells [51]. In murine experiments, NSG mice carrying human *BRAF*^V600E^ CD34^+^ cells exhibit RO+ MS LCH-like disease (involving liver and spleen) and expansion of CD11c^+^CD14^+^ cells in peripheral blood [65]. The expression of *BRAF*^*V600E*^ in bone marrow dendritic cell progenitors (*BRAF*^*V600E*^ expressed under the CD11c promoter, *BRAF*V600E ^CD11c^) induces aggressive multisystem disease and dramatically elevates blood circulating dendritic cell precursors [45]. Taken together, the expression of *BRAF*^V600E^ in CD34^+^ hematopoietic cells could lead to the development of LCH.

*BRAF*^V600E^ mutation seems to influence the proportion of circulating MNPs. Pediatric LCH patients have a higher CD14^+^ monocytes level whereas a lower pDCs level than healthy control, and the lower pDCs level was associated with higher cell-free *BRAF*^V600E^ level and more severe disease [48].

In terminally differentiated LCH cells, the senescence state of progenitor cells persisted. *BRAF*^V600E^ mutation also leads to LCH cells isolated from lesions expressed senescence signature (including *CDNK2A*, *CDKN2B*, and *CDKN2C*) that result in low Ki-67 of those cells [65]. This is validated by the low Ki-67 in LCH lesions, the Ki-67 positive LCH cells distributed scattered in LCH lesions, taking only about 1.9% of LCH cells, no evidence of *in situ* proliferation of LCH cells in lesions has been identified [45, 66–68]. The senescence state also contributes to high SASP transcript level (MMP1, MMP3, MMP9, MMP13), and may lead to production of inflammatory cytokines such as IL-1, IL-6, and MMPs [65].In addition, The *BRAF*^V600E^ mutation and a senescence state are also associated with anti-apoptosis activity, high B-cell lymphoma 2 like 1 (BCL2L1, BCL-XL homologue) expression was detected in human LCH cells [67]. ERK1/2 activation also inhibits pro-apoptotic MAPK effects in dendritic cells [69] (Figure 3D(a)).

Continuous activation of the MAPK pathway may lead to abnormal entrapment of LCH cells in lesions. In physiological situations, resting skin Langerhans cells express C–C chemokine receptor type 6 (CCR6) and stayed in the epidermis by contact with C-C motif chemokine ligand (CCL) 20 (CCL20) expressed by epidermal keratinocytes [70]. Under inflammation, Langerhans cells become mature, downregulate CCR6 expressions whereas upregulate CCR7 expressions, and migrate to lymphoid tissues by anchoring with the ligand of CCR7 (CCL19 and CCL21) [57]. Contradictory CCR7 expression levels were presented in human LCH lesions. Annels et al. revealed that in LCH lesions, LCH cells expressed CCR6 and are absent of CCR7 [71]. Quispel et al showed that 7 of the 25 LCH lesions were CCR6^-^CCR7^+^, and the others were [72] CCR6^+^CCR7^-^. Whereas Fleming et al. argued that CCR7 and CCR6 were present concurrently [73].Murine experiments revealed that the dendritic cells in the *BRAF*V600E^CD11c^ mice showed dramatically reduced migration ability into draining lymph nodes due to significantly downregulated expression of CCR7, even under the stimulation of TNFα and IL-1, compared with the normal control skin Langerhans cells [67] (Figure 3D(b)). Unlike other cells that utilize the MAPK pathway for the regulation of survival and proliferation, dendritic cells mainly utilize CCR7 and the downstream MAPK pathway for controlling migration to lymph nodes but utilize the PI3K/Akt pathway for survival control [69]; thus, the continuous activation of the MAPK pathway in dendritic cells may play a negative feedback role that downregulates CCR7 expression in LCH cells (Figure 3D(b)). Further experiments are needed to evaluate the CCR7 expression level in human LCH lesions. Moreover, Quispel et al also revealed that C-X-C motif chemokine receptor 4 (CXCR4) is generally expressed by LCH cells, CD1a^+^CXCR4^+^ cells could also be detected in the blood, and C-X-C motif chemokine ligand 12 (CXCL12, the ligand of CXCR4) is detected in most LCH lesions, thus the CXCR4-CXCL12 interaction may contribute to LCH cells homing and trapping in lesions [72].

These results reveal that the accumulation of LCH cells in lesions is due to the entrapment and anti-apoptosis of tumor cells in the lesion rather than *in situ* proliferation.

## The pathological function of histiocytes and the inflammatory microenvironment in LCH

In lesions, in addition to LCH cells, multiple clusters of immune cells exist, including T cells, neutrophils, eosinophils, B cells, plasma cells, myeloid-derived suppressor cells, monocytes, macrophages [1, 51, 74], and multinucleated giant cells (MGCs) [50, 75]. Whether these cells are innocent bystanders in the pathogenesis of the disease has not been substantially determined.

### LCH cells are functionally abnormal antigen-presenting cells

The lesions in mice having *BRAF*^V600E^ hematopoietic cells [65], *BRAF*V600E^CD11c^ mice [62] and NSG mice [65] carrying human *BRAF*^V600E^ CD34^+^ cells present an inflammatory background similar to that in humans, which indicates that the inflammatory background is seeded by tumor dendritic cell progenitors. A transcriptomic study revealed that LCH cells express *SPP1*, which encodes osteopontin that could recruit T cells to sites of inflammation [45]. The senescence phenotype of LCH cells promote inflammatory cytokine production and may contribute to inflammatory cells recruitment in lesions [65]. LCH cells have some antigen presentation function but are predominantly dysfunctional. In a physiological state, interactions between antigen-presenting cells (APCs) and T cells are mediated by membrane-bound costimulatory receptor and receptor ligand pairs, such as CD40-CD40 ligand (CD40L) and CD152-CD80/CD86, which bidirectionally activate T cells and APCs [76, 77], enhance cytokine production by T cells and APCs (e.g., IFN-γ and TNF-α), and further recruit other immune cells. In LCH lesions, there was prominent expression of CD40 on LCH cells and CD40L on T cells [78–80]. However, despite the universal expression of CD40L by T cells, almost all LCH cells do not express CCR7, CD83, CD86 or lamp3 *in vivo* and can poorly stimulate T cells *in vitro*, suggesting that they represent functionally immature dendritic cells [71, 80] . LCH cells carrying the *BRAF*^*V600E*^ mutation are also unable to present abnormal neoantigens on human leukocyte antigen (HLA) class I molecules, which hampers their ability to stimulate CD8^+^ T cells and may help LCH cells avoid immune surveillance [81] . As mentioned above, the senescence phenotype and JAG2 mediated Notch activation would stimulate MMP production by LCH cells, which may contribute to tissue destruction in LCH lesions [46, 65].

### The microenvironment may contribute to the in situ differentiation of precursor cells

Colony stimulating factor 1 (CSF1) is the ligand of CSF1R and is universally expressed in LCH lesions [42]. In cell experiments, CSF1 induced the differentiation of CD1a^+^CD207^+^ cells from CD34^+^ progenitors, and blockade of the CSF1/CSF1R signaling pathway with BLZ945 reduced differentiation [42]. Thus, CSF1 expression in the lesion may help to further recruit CSF1R+ progenitor cells and aid the *in situ* differentiation of progenitor cells. CSF1R activation by CSF1 can also activate the downstream MAPK pathway, regardless of the *BRAF* mutation state [42].

### T cell subsets are disproportional and dysfunctional in LCH lesions

The components of T cells are abnormal and dysfunctional in LCH patients. Recently, utilizing mass cytometry, Sengal *et al.* substantially studied the T cell subsets in LCH lesions [75]. Independent of the *BRAF* mutation state, the number of CD4^+^ T cells was significantly larger than that of CD8^+^ T cells (74% vs. 21%), and the frequency of Tregs among CD4^+^ T cells was significantly higher than that in normal tissue controls [75]. The proportion of CD8^+^ T cells expressing a higher level of PD-1 receptor, T cell immunoglobulin and mucin domain-containing protein 3 (TIM-3), and lymphocyte activation gene 3 protein (LAG-3) was significantly higher in the lesions than in the peripheral blood of the same patient or that in the control [75]. These cells secreted significantly lower levels of granzyme B and perforin than PD-1^-^TIM-3^-^LAG-3^-^CD8^+^ T cells, indicating an exhausted phenotype [75]. LCH cells expressed significantly higher levels of PD-L1 [75, 82] and PD-L2 [75], which play an important role in inhibiting CD8^+^ T cells, than healthy skin cells. When the cells were treated with anti-PD-1 antibodies, the effector function of CD8^+^ T cells was rescued [75]. These results were validated in *BRAF*V600E^CD11c^ mice [75]. PD-1 and PD-L1 expression patterns were also proved in 6 pulmonary LCH by immunostaining [83]. A high PD-L1 positive rate of lymphocytes was suggested by Hashimoto et al, but they did not co-stain PD-L1 with LCH cells [84]. These findings indicate an important function of the PD-1 and PD-L1 interaction in preventing CD8^+^ T cells from performing their normal function in LCH lesions [75] (Figure 3C(b)). A transcriptomic study comparing CD3^+^ cells from LCH lesions and peripheral blood revealed that lesion CD3^+^ T cells showed an activated regulatory T cell (Tregs) feature (*FOXP3*, *CTLA4*, and *SPP1*) [45]. Further investigation regarding the CD4^+^ T cell subtypes revealed that nearly 20% of the T cells are CD4^+^CD25^high^FoxP3^high^ regulatory T cells, and these Tregs are located adjacent to LCH cells [66, 85] . These Tregs show competent suppressive activity when isolated [75, 86]. A large proportion of Tregs express CD56, and the proportion of CD56^+^ Tregs in the lesions of active disease patients was increased compared with that in patients with nonactive disease, whereas there was a decreased proportion of CD56^+^ Tregs in peripheral blood, which indicates that Tregs are recruited into the lesion from peripheral blood [86]. Tregs express inducible costimulatory factor (ICOS) and interact with adjacent LCH cells that express ICOS ligand [66, 87], which may play an important role in the induction and expansion of Tregs [87, 88] (Figure 3C(a)). TGF-β and IL-10 are mainly produced by LCH cells and macrophages, as well as Tregs [86, 87]. Dendritic cells are specialized cells that together with TGF-β can assist the differentiation of FoxP3^-^ precursor T cells from the blood into Foxp3^+^ Treg cells [89]. Thus, in addition to aiding the accumulation of naturally occurring Tregs (nTregs), LCH cells may also promote the differentiation of activated Tregs (aTregs). Both types of Tregs construct a suppressive microenvironment that protects LCH cells from immune surveillance. In addition, unconventional T cell subsets were also altered in LCH lesions, the proportion of mucosal-associated invariant T (MAIT) cells of whole T cells is significantly decreased in LCH patients’ blood compared with that in healthy control, but the clinical significance had not been determined yet [90].

### Beyond T cells

In addition to T cell compartments, eosinophils are also attracted into lesions mainly via C-C motif chemokine ligand 5 (CCL5) produced by LCH cells [71] and IL-5 produced by T cells [91] (Figure 3C(c)), but the exact role of eosinophils in the pathogenesis of LCH lesions is unknown. MGCs that were formed under the stimulation of GM-CSF, receptor activator of nuclear factor κ-B ligand (RANKL), and IL-17 in vitro [92], express MMP9 [50] and may perform a destructive role in the lesion [93].

These evidence support the pathological functional role of LCH cells as functionally attenuated antigen-presenting cells that are trapped in the lesion due to their inability to express CCR7 but recruit other immune cells into and maintain the lesion.

## No biomarker for risk stratification has been identified in LCH

Despite the recurrent MAPK pathway mutations in LCH, the relationships between mutation type and clinical manifestations, response to chemotherapy, and outcomes are uncertain. In a cohort containing 100 pediatric patients (age range 0–9.3 years), the expression of *BRAF*^*V600E*^ in CD207^+^ cells in LCH lesions did not correlate with clinical risk groups, risk of central nervous system involvement, or diabetes insipidus state but was associated with a higher risk of recurrence [6, 62]. In another cohort with 315 pediatric patients, including 173 patients with the *BRAF*^*V600E*^ mutation, the *BRAF*^V600E^ mutation was associated with higher-risk manifestations and poorer response to chemotherapy [94]. In a Chinese cohort with 73 adult LCH patients, *BRAF* indel mutations were associated with shorter event-free survival than other *BRAF* mutations [25].

## Efficacy of kinase signaling pathway inhibition

### MAPK pathway inhibition

Since the discovery of recurrent *MAPK* pathway mutations in LCH and the confirmation of *MAPK* pathway activation (ERK phosphorylation) in LCH lesions [5–7], *MAPK* pathway inhibitors, which have been used in the treatment of other tumors, such as melanoma, have been used to treat histiocytes in patients and early-phase trials. BRAF inhibitors are categorized into αC-in and αC-out based on the BRAF conformation that they bind with [95]. αC-out inhibitors such as vemurafenib and dabrafenib, bind to the αC helix in OUT position, preventing it from activating [96]. However, αC-out inhibitors could not bind BRAF that is already in αC-IN conformation or BRAF dimer, so Class II *BRAF* mutations and β3-αC3 deletions were resistant to this class of inhibitor [20, 29, 96]. The αC-in inhibitors could bind to BRAF dimer and BRAF in αC IN state, and could be effective in mutations that are resistant to αC-out inhibitors [96] (Figure 2). In LCH patients bearing the *BRAF*^V600E^ mutation, treatment with αC-out RAF inhibitors (vemurafenib and dabrafenib) has been proven to be effective, showing rapid and effective regression of lesions and constitutional symptoms, regardless of the response to previous treatments [36, 48, 64, 97–109]. The *BRAF*^N486_P490indel^ mutation, is insensitive to αC-out RAF inhibitors [28, 29], but responds to MEK inhibitors (trametinib) [24] and may respond to αC-in RAF inhibitors (such as sorafenib) [29]. In addition, patients carrying RAF-dependent MAP2K1 mutations might also be sensitive to RAF inhibitors [38].

MEK inhibitors (e.g., trametinib and cobimetinib) have also shown effectiveness in patients with various histiocytosis, including patients carrying BRAF^V600E^ mutations and other mutations in the MAPK pathway [36, 104, 106, 110–115]. However, RAF-independent mutations are resistant to allosteric MEK inhibitors (which bind to an inactive form of MEK) [38], and one patient with the *MAP2K1*^*p.L98_K104*>*Q*^ mutation showed resistance to the MEK inhibitor trametinib [34]. For such patients, ATP-competitive MEK inhibitors might be effective (all of which are under clinical investigation) [38] (Figure 3B(b)).

### Mechanisms of MAPK pathway inhibitors on LCH cells

Cell and animal experiments have provided evidence about the mechanisms of *MAPK* pathway inhibitor treatment in histiocytosis. Phosphorylation of ERK1/2 was inhibited by vemurafenib (a RAF inhibitor) or U0126 (a MEK inhibitor) in *BRAF*^*V600E*^ CD207^+^ cells or transfected HEK293 cells, whereas it was only inhibited by MEK inhibitors in *MAP2K1*-mutated CD207^+^ cells, transfected HEK293 cells [6], and 3T3 cells [24]. Culturing *BRAF*^*V600E*^ CD207^+^ dendritic cells from human skin lesions with vemurafenib or trametinib (a MEK inhibitor) induces a significant increase in CCR7 expression and a decrease in BCL2L1 expression [67]. Treating *BRAF*V600E ^CD11c^ mice with a MEK inhibitor at 1 mg/kg/d restores the expression of CCR7 on dendritic cells and improves LCH-like lesions and survival [67, 75]. The *BRAF*^*V600E*+^ cells persist in peripheral blood after MAPK inhibitor treatment, though this persistence was not correlated with disease activity and or clinical responses, whether it would bury the scourge of disease relapses is still unknown [64, 104, 116, 117]. Thus, MAPK inhibitors seem to modulate the differentiation and function of tumor LCH cells rather than eradicate tumor cells or precursors like chemotherapy. Evseev et al. reported 9 infants receiving vemurafenib and chemotherapy (e.g. cytarabine, vinblastine) simultaneously as salvage treatment, 8 of them showed response without toxicity [117]. However, combination therapy did not eradicate the disease, five of the 8 patients soon relapsed after discontinuing vemurafenib, and need vemurafenib maintenance therapy [117]. Additional clinical trials evaluating the efficacy of the combination of MAPK inhibitors and chemotherapy are encouraging.

Proper maintenance treatment is also a critical issue since rapid disease reactivation occurs after drug discontinuation [97, 106]. Although reintroduction of the drug could still be effective, acquired resistance to *MAPK* inhibitors can occur at an alarming rate; this phenomenon has been observed in melanoma, although it has not been observed in histiocytosis. There is also a risk of paradoxical activation of other signaling pathways with RAF inhibitor treatment. When treating patients who bear kinase mutations other than *BRAF*^*V600E*^, vemurafenib treatment results in an increase in blood cell counts, especially in patients carrying the *JAK2*^V617F^/*IDH2*^R140Q^ mutation. Treatment with vemurafenib causes paradoxical stimulation of Janus kinase 2 (JAK2), resulting in an increase in monocytes [118]. Thus, before kinase inhibitor treatment, it is important to evaluate the mutational landscape of these patients.

### Other kinase pathway inhibitors

Beyond MAPK pathway inhibitors, the efficacy of the pan-AKT inhibitor afuresertib in the treatment of LCH has been evaluated in a phase II trial [11]. In one phase II trial, two of the 17 patients included in the study had the *BRAF*^V600E^ mutation, while others were *BRAF* wild type. The overall response rate was approximately 30%. There was no apparent relationship between *BRAF* mutation status and treatment response. The response rate did not meet the futility or efficacy criteria for the study, and the treatment did not show better efficacy than other regimens currently available; thus, further development of this monotherapy for LCH was not recommended [11]. Inhibitors of other possible treatment targets of LCH, such as PI3K inhibitors and CSF1R inhibitors, could be promising for LCH treatment. Abekoon *et al.* recently showed a refractory ECD patient carrying *CSF1R*^R549_E554delinsQ^ mutation treated with pexidartinib (a CSF1R inhibitor) showed sustained and complete remission [10]. Further clinical trials in LCH are needed.

### Other potential therapies

#### Combination therapy with PD-1/PD-L1 inhibitors

Recently, Sengal et al. reported a preclinical experiment that combined a BRAF inhibitor and an anti-PD-1 antibody in the treatment of LCH model mice. Mass cytometry and immunohistochemical analyses of the cell compartments of mouse lungs and livers revealed that after BRAF inhibitor monotherapy, myeloid CD11b^+^ cells (myeloid dendritic cell progenitors) decreased, whereas CD4^+^ and CD8^+^ infiltrating T cells increased [75]. Therefore, the researchers proposed that the inflammatory background of the tumor lesion could be another treatment target. Culture of CD8^+^ T cells with anti-PD-1 antibodies rescued the cytotoxic function of those cells [75] (Figure 3C(b)). Treating *BRAF*V600E^CD11c^ mice with anti-PD-1 antibodies significantly reduced the disease burden, showing a comparable effect to MEK inhibitors [75]. However, the number of dendritic cell precursors and lymphoid infiltration were not decreased in the lungs and livers of mice after a single application of anti-PD-1 antibody treatment [75]. MEK inhibitors combined with anti-PD-1 treatment showed a synergistic effect, with a decrease in infiltrating myeloid cells and lymphoid cells, as well as a restored function of CD8^+^ T cells, but survival analysis could not be performed [75]. However, anti-PD-L1 antibody treatment alone had a minimal effect [75] . In melanoma mice bearing similar MAPK pathway mutations, treatment with PD-1 inhibitors increased the CD8^+^ T cell population and enhanced the antitumor effect of MAPK pathway inhibitors [119]. These preclinical studies provided evidence for further clinical trials of agents targeting tumor cells and the inflammatory background simultaneously.

#### Therapy targeting the senescent cells

The SASP in senescent cells is considered due to continuous activation of the mTOR pathway [120]. In in vitro cell experiments, inhibition of the mTOR pathway by rapamycin (a mTOR pathway inhibitor) succeeded in reducing inflammatory cytokines production and the differentiation potential toward MNPs in *BRAF*^V600E^+ CD34+ cells [65]. In mice carrying *BRAF*^V600E^ hematopoietic cells, rapamycin reduces the MNPs in the bone marrow, improves the organomegaly and inflammatory infiltration of organs involved, though the apoptosis of *BRAF*^V600E^+ cells did not increase and the possibility that rapamycin may have a direct effect on infiltrating T cells could not be ruled out [65]. Direct targeting at the senescent cells would also be a promising strategy. Treating LCH cells with ABT-263 (a BCL-XL inhibitor) could increase their apoptosis [67], and treating mice carrying *BRAF*^V600E^ hematopoietic cells with ABT-263 that eliminate senescent cells could clear *BRAF*^V600E^+ cells and improve the clinical manifestations of those mice [65].

## Conclusion

In conclusion, mutations that cause continuous MAPK pathway activation play a fundamental role in the pathogenesis of Langerhans cell histiocytosis, affecting processes including proliferation, functional attenuation, entrapment, and inflammatory background formation. MAPK pathway inhibition alone or in combination with immunotherapy targeting the inflammatory background is a promising treatment strategy for controlling Langerhans cell histiocytosis, especially for patients who had relapse/recurrent disease after front-line therapy. Whenever MAPK pathway inhibitors are considered, MAPK pathway mutation types should be carefully evaluated to determine which specific categories of MAPK pathway inhibitors are ideal for patients.

## Data Availability

Not applicable.

## Abbreviations

AKT: Protein kinase B
APC: Antigen presenting cell
aTregs: Activated Tregs
BCL2: B-cell lymphoma 2
BCL2L1: B-cell lymphoma 2 like 1
BCL-XL: B-cell lymphoma-extra large
BMP7: Bone morphogenetic protein-7
CCL20: C–C motif chemokine ligand 20
CCR6: C–C chemokine receptor type 6
CCR7: C–C chemokine receptor type 7
cDC: Conventional dendritic cell
CR: Conserved regions
CSF1: Colony stimulating factor 1
CSF1R: Colony stimulating factor 1 receptor
CXCL12: C–X–C motif chemokine ligand 12
CXCR4: C–X–C motif chemokine receptor 4
ECD: Erdheim-Chester disease
ERK: Extracellular-signal regulated kinase
GM-CSF: Granulocyte macrophage colony-stimulating factor
(G)MODP: Granulocyte/macrophage/osteoclast/dendrite cell progenitor
GMP: Granulocyte–macrophage progenitor
HLA: Human leukocyte antigen
ICOS: Inducible costimulatory factor
JAK2: Janus kinase 2
LAG-3: Lymphocyte activation gene 3 protein
LCH: Langerhans cell histiocytosis
LMPP: Lymphoid-primed multipotent progenitor
MAIT cells: Mucosal-associated invariant T cells
MAPK: Mitogen-activated protein kinase
M-CSF: Macrophage colony-stimulating factor
MEK: Mitogen-activated protein kinase kinase
MGC: Multinucleated giant cell
MMP: Matrix metalloproteinase
MNP: Mononuclear phagocytes
MS: Multisystem disease
mTOR: Mammalian target of rapamycin
NSG: Nonobese diabetic severe combined immunodeficient gamma
NSGS: Nonobese diabetic severe combined immunodeficient gamma with both IL2Rgamma knockout and transgenic cytokine expression (with transgenic expression of human IL-3, stem cell factor, and granulocyte–macrophage colony-stimulating factor)
nTregs: Naturally occurring Tregs
PBMC: Peripheral blood mononuclear cell
pDC: Plasmacytoid dendritic cell
PI3K: Phosphatidylinostitol-3-kinase
PLCH: Single-system pulmonary disease
RAF kinase: Rapidly accelerated fibrosarcoma kinase
RANKL: Receptor activator of nuclear factor κ-B ligand
RBD: RAS GTP-binding self-regulatory domain
RDD: Rosai-Dorfman disease
RO: Risk organ
SAβGal: Senescence-associated β-galactosidase
SASP: Senescence-associated secretory phenotype
SS-s: Single-system unifocal disease
SS-m: Single-system multifocal disease
ssGSEA: Single-sample gene set enrichment analysis
TGF-β: Transforming growth factor-beta
TIM-3: T cell immunoglobulin and mucin domain-containing protein 3
Tregs: Regulatory T cells
TSLP: Thymic stromal lymphopoietin
